# A Deep Learning Approach for Missing Data Imputation of Rating Scales Assessing Attention-Deficit Hyperactivity Disorder

**DOI:** 10.3389/fpsyt.2020.00673

**Published:** 2020-07-17

**Authors:** Chung-Yuan Cheng, Wan-Ling Tseng, Ching-Fen Chang, Chuan-Hsiung Chang, Susan Shur-Fen Gau

**Affiliations:** ^1^ Institute of Biomedical Informatics, National Yang-Ming University, Taipei, Taiwan; ^2^ Department of Psychiatry, National Taiwan University Hospital and College of Medicine, Taipei, Taiwan; ^3^ Child Study Center, Yale University School of Medicine, New Haven, CT, United States; ^4^ Graduate Institute of Brain and Mind Sciences, and Graduate Institute of Clinical Medicine, College of Medicine, National Taiwan University, Taipei, Taiwan

**Keywords:** ADHD, oppositional behavior, missing data imputation, deep learning, rating scale, continuous performance test, classifications

## Abstract

A variety of tools and methods have been used to measure behavioral symptoms of attention-deficit/hyperactivity disorder (ADHD). Missing data is a major concern in ADHD behavioral studies. This study used a deep learning method to impute missing data in ADHD rating scales and evaluated the ability of the imputed dataset (i.e., the imputed data replacing the original missing values) to distinguish youths with ADHD from youths without ADHD. The data were collected from 1220 youths, 799 of whom had an ADHD diagnosis, and 421 were typically developing (TD) youths without ADHD, recruited in Northern Taiwan. Participants were assessed using the Conners’ Continuous Performance Test, the Chinese versions of the Conners’ rating scale-revised: short form for parent and teacher reports, and the Swanson, Nolan, and Pelham, version IV scale for parent and teacher reports. We used deep learning, with information from the original complete dataset (referred to as the reference dataset), to perform missing data imputation and generate an imputation order according to the imputed accuracy of each question. We evaluated the effectiveness of imputation using support vector machine to classify the ADHD and TD groups in the imputed dataset. The imputed dataset can classify ADHD vs. TD up to 89% accuracy, which did not differ from the classification accuracy (89%) using the reference dataset. Most of the behaviors related to oppositional behaviors rated by teachers and hyperactivity/impulsivity rated by both parents and teachers showed high discriminatory accuracy to distinguish ADHD from non-ADHD. Our findings support a deep learning solution for missing data imputation without introducing bias to the data.

## Introduction

Attention-deficit/hyperactivity disorder (ADHD) is a common childhood-onset neuropsychiatric disorder with developmentally inappropriate inattention, hyperactivity, and impulsivity (1, 2). The current epidemiological prevalence rate of ADHD is 9.4% in the USA (3) and 8.7% in Taiwan (4). Children and adolescents with ADHD are at increased risk for academic underachievement (5), behavioral problems at school (5, 6), impaired peer (6–8) and parent-child (9, 10) relationships, emotional dysregulation (11, 12), and oppositional and conduct problems (12, 13). Many individuals with ADHD continue to have ADHD symptoms in adulthood (14), suffer from comorbid psychiatric conditions (15), and have persistent executive dysfunctions (16, 17), social impairments (18), and reduced life quality (18) and health conditions (14). Given its high prevalence and long-term impairment, there is a pressing need for early detection, diagnosis, and intervention of ADHD in youth population. Although clinical diagnosis has been recognized as the gold standard for ascertaining ADHD in clinical practice, attention tests and standardized rating scales measuring ADHD and related symptoms are often used to screen for potential cases of ADHD (19), assist in diagnosis (20), and monitor symptom changes over time (21, 22) or in response to treatment (23–27).

Of the core symptoms of ADHD, hyperactivity and impulsivity are more readily observable than attention problems (19, 28). Given this, rating scales covering inattention symptoms may not adequately capture the attention deficits, especially when rating scales are completed by informants other than the subjects themselves. Thus, objective instruments that measure a wide range of attention performance could be helpful in this case. Due to its simplicity and comprehensive coverage of domains in attention and impulsivity, the continuous performance test (CPT) has been widely used in clinical research to aid in assessments of ADHD (29, 30). CPT is designed to engage subjects in a monotonous and repetitive task over an extended time (usually more than 10 min), e.g., letters “A–Z” appear sequentially on the screen and subjects are instructed to respond if any letter other than the target letter (e.g., “X”) shows up on the screen. This task is simple but requires vigilance and sustained attention. Past research has documented that children with ADHD performed worse on CPT than controls (31, 32), despite some concerns about its psychometric properties and ecological validity (33).

Clinical interview with the child and their caregivers is the gold starndard for diagnosing ADHD. Direct observations of the child, neuropsychological and cognitive assessment (e.g., with CPT), and the use of self-administered questionnaires completed by parents and/or teachers (31, 33) can sometimes be helpful to aid in the diagnosis of ADHD. Questionnaires and rating scales are a cost-effective and efficient way to screen for ADHD and related symptoms. The Chinese versions of several internationally recognized ADHD instruments (e.g., the Conners Rating Scales and the Swanson, Nolan, and Pelham, Version IV Scale) have been prepared for this purpose, and their psychometric properties had been established in our previous work (19, 21, 22, 34). Whenever feasible, teachers’ reports should be included, as they provide valuable information about the child’s behavior in relation to other same-age peers. Also, teachers may be more likely than parents to identify attention problems in the classroom because they have more opportunities to observe children doing classroom work and tasks that require sustained attention and concentration (35). Despite the low agreement between parent and teacher reports on ADHD symptoms in western studies (35, 36) and our work (37, 38), it is crucial to integrate reports from different informants. Because parents and teachers see the child in different contexts, they each provide unique, valuable cross-context information about the child, which is important when evaluating the cross-context diagnostic requirement of ADHD. Therefore, in this study, we included data from both parent and teacher reports of ADHD symptoms, in addition to clinical interviews conducted by clinicians/psychiatrists as well as children’s performance on the CPT.

A common methodological issue for data collection in a large survey-based or epidemiology study is missing data (39–43). There are several approaches to handling missing data prior to data analysis (44–47). First, the complete-case approach (listwise deletion), which only includes cases with complete data for the analysis, is the simplest to deal with missing data. However, it significantly reduces power for the analysis and can introduce biases if the excluded subjects are systematically different from those included. Second, missing data can be replaced with the mean of the available cases. This is an easily applied approach, but it reduces the data variability and underestimates both the standard deviations (SD) and variances (45, 48). Third, missing values can be imputed using a regression model where available data from other variables are used to predict the value of a particular variable for which data are missing. However, using regression imputation overestimates the correlations between target variable and explanatory variable and also underestimates variances and covariances (48). Fourth, the hot-deck imputation approach, commonly used in surveys, can be used to identify the respondents who share similar characteristics as the non-respondents and then impute missing data from the resembling respondents (49). Fifth, the inverse probability weighting is a method to calculate statistics of a population different from that in which the data collected. Through estimating sampling probability, this method can be used to expand the weight for subjects who have a significant degree of missing data (50). Lastly, multiple imputations, based on multiple regressions, imputes missing data by creating several different plausible imputed datasets and appropriately combining results obtained from each of them (51). Interested readers are referred to the work of Burton and Altman (44), Eekhout et al. (45), Wood et al. (46), and Pigott (47) for the reviews on different methods for handling missing data.

Most of these proposed imputation techniques may bias study results (49, 52). Inverse probability weighting and multiple imputation have been shown to work well when assumptions of missing completely at random (MCAR) and missing at random (MAR) hold (38, 53, 54). Despite great efforts to solve the missing data problem, none of the abovementioned approaches are fully satisfactory. An approach that does not bias the estimated parameters is needed. In recent years, researchers have started to apply machine learning to missing data imputation, reporting that machine learning methods outperform traditional statistical methods (e.g., mean imputation, hot-deck, multiple imputations) in handling missing data, resulting in better prediction accuracy of patient outcome (55).

Deep learning, a branch of machine learning methods based on artificial neural network (ANN), has been proposed in the early 1980s (56) but limited in use because of the cost in time and computational resources given the hardware constraints at the time. With the availability of large labeled datasets and Graphics Processing Units (GPUs) which greatly accelerate the computing process in deep learning frameworks, deep learning has started to gain popularity in recent years (57). Deep learning has been applied to various domains such as image classification, speech recognition, and language processing, often outperforming traditional machine learning methods such as support vector machine (SVM). The ability of deep learning to infer abstract, high-level representations makes it a promising approach for the prediction of diagnosis, prevention, treatment, and prognosis of mental illness (58, 59). A few studies have used deep learning to classify disorders, including ADHD, Alzheimer’s disease, and dementia (60–64). Deep learning-based approaches have also been shown to perform well as a missing data imputation method in large, high-dimensional datasets (65, 66).

In this study, we propose an approach based on deep learning to impute missing data in ADHD questionnaires. To the best of our knowledge, this work is the first to apply deep learning to clinical data imputation in ADHD. We combined multiple samples from our previous studies to increase the total sample size (N=1220) and used a deep learning approach to impute the missing data in parent- and teacher-rated ADHD scales. We expect deep learning to be able to impute missing values and generate a complete imputed dataset that resembles the original complete dataset (referred to as the reference dataset) as closely as possible in its ability to distinguish ADHD from TD children. In addition, through the process of this deep learning approach, we can rank the questions of the rating scales in terms of the ability of the machine to learn from the data and to predict the missing values accurately. We hypothesized that questions assessing hyperactivity-impulsivity behaviors, particularly from teacher reports, would have high imputation accuracy and discriminating ability based on previous studies suggesting that these symptoms are observable (67) and that teachers may have more opportunities to observe ADHD-related behaviors such as oppositional defiant symptoms than parents do (35).

## Materials and Methods

### Sample and Procedures

The sample consisted of 799 youths with a clinical diagnosis of ADHD (689 boys, 86.2%) according to DSM-IV diagnostic criteria and 421 typically developing (TD) youths (343 boys, 81.5%). The sample came from two separate studies – a longitudinal study of adolescent outcomes in children with ADHD aged 11-16 years (192 ADHD and 142 TD) conducted during 2006-2009 and a genetic, treatment, and imaging study of drug-naïve children and adolescents with ADHD aged 6-18 years (607 ADHD and 279 TD) conducted during 2007-2015. Youths with ADHD were recruited from the child psychiatric clinic in National Taiwan University Hospital (NTUH), Taipei, Taiwan. The TD youths without a lifetime diagnosis of ADHD were recruited from the same school districts as youths with ADHD *via* the help of school principals and teachers. All the participants and their parents were interviewed using *the Chinese version of the Kiddie Epidemiologic Version of the Schedule for Affective Disorders and Schizophrenia* (2) to confirm the presence or absence of ADHD diagnoses and other psychiatric disorders. Participants with major medical conditions, psychosis, depression, autism spectrum disorder, or a Full-Scale IQ score less than 70 were excluded from the study.

Participants’ IQ and attention were assessed using the Weschler Intelligence Scale for Children-3^rd^ edition (WISC-III) (68) and Conner’s CPT (CCPT) (69), respectively. Participants’ parents and teachers completed questionnaires assessing the core symptoms of ADHD and related symptoms such as oppositionality by using the Chinese version of the Conners’ parent and teacher rating scales-revised: short form (CPRS-R:S/CTRS-R:S) (19, 34) and the Chinese version of the Swanson, Nolan, and Pelham, version IV scale (SNAP-IV) reported by parents (22) and teachers (21). These scales have been widely used in the screening for ADHD or measuring the intervention/treatment effect in clinical, community, and research settings (6, 19, 32, 70–76). Given that symptoms of oppositional defiant disorder (ODD) are included in all these scales and ODD symptoms are highly associated with ADHD and easily observed by teachers and parents (77), we included ODD items in the analyses and further hypothesized that ODD symptoms reported by teachers can distinguish ADHD from non-ADHD. These studies were approved by the Research Ethics Committee of National Taiwan University Hospital, Taipei, Taiwan (Approval numbers: 200612114R, 200812153M, 9361700470; ClinicalTrials.gov number: NCT00529906, NCT00916786, NCT00417781) before study implementation. The data were collected after the participants, their parents, and their teachers provided written informed consent.

### Measures

#### The Chinese Version of the Kiddie Epidemiologic Version of the Schedule for Affective Disorders and Schizophrenia (Chinese K-SADS-E)

The K-SADS-E is a semi-structured interview scale for a systematic assessment of both past and current mental disorders in children and adolescents. The Chinese version of K-SADS-E was developed by the Child Psychiatry Research Group in Taiwan (2, 78). To ensure that the DSM-IV diagnostic criteria and language were culturally appropriate and sensitive for the Taiwanese child and adolescent populations, the development of this instrument included two-stage translation and modification of several items with psycholinguistic equivalents. This scale has been widely used in child and adolescent clinical research in Taiwan [e.g., (75, 79, 80)].

#### The Conners’ Continuous Performance Test (CCPT)

The CCPT is a 14-minute, non-X type test design for ages 6 and up (81). Participants are asked to press the space bar when a character (target) shows up on the screen, except when the *X* (non-target) shows up. There are six blocks in CCPT, with three sub-blocks in each block. Each sub-block has 20-letter presentations. The sub-blocks differ in Inter-Stimulus Intervals (ISIs) of 1, 2, and 4 s, and the sequence of ISI conditions presents randomly. There are 12 indices covering different domains of CCPT performances: (1) Omission errors: the number of times the target is missed; (2) Commission errors: the number of times *X* is hit; (3) Variability: intra-individual variability in reaction time; (4) Perseveration: a reaction time less than 100 ms; (5) Detectability: the ability to distinguish X from non-X letters; (6) Reaction Time (RT): the period of time between the presentation of the stimulus and the response; (7) Hit RT Standard Errors (Hit RT SE): consistency of response time; (8) Response Style: a function of the ratio of hit target to hit non-target stimuli; (9) Hit RT change by blocks (Hit RT BC): the slope of change in RT over six blocks as the test progresses; (10) Standard error of Hit RT change by blocks (Hit RT SE BC); (11) Hit RT changed by ISIs (Hit RT ISI Change); (12) Standard error of Hit RT changed by ISIs (Hit SE ISI Change). Indices of CCPT can be grouped into several dimensions (82): (1) Focused attention: RT, Hit RT SE, detectability, and omission errors; (2) Sustained attention: Hit RT BC and Hit RT SE BC; (3) Hyperactivity/impulsivity: commission errors, RT, response style, and perseverations; (4) Vigilance: Hit RT ISI Change and Hit SE ISI Change. We used all 12 indices in this study.

#### The Chinese Version of the Swanson, Nolan, and Pelham, Version IV Scale (SNAP-IV)

The Chinese SNAP-IV form is a 26-item scale rated on a 4-point Likert scale with 0 for not at all (never), 1 for just a little (occasionally), 2 for quite a bit (often), and 3 for very much (very often). There are nine items for inattention (item 1–9) and nine items for hyperactivity/impulsivity (item 10-18) of the core symptoms of ADHD and eight items for the ODD symptoms according to the DSM-IV symptom criteria for ADHD and ODD (77). The psychometric properties of Chinese SNAP-IV Parent (22) and Teacher Form (21) have been established in Taiwan, and the scales have been frequently used to assess ADHD and ODD symptoms in clinical and research settings [e.g., (32, 73–76)].

#### The Chinese Version of the Conners’ Parent and Teacher Rating Scales-Revised: Short Form (CPRS-R:S/CTRS-R:S)

The Conners’ Rating Scales (CRS), developed in 1969, have been widely used for screening and measuring ADHD symptoms (83–86). We used the short version in this study – the 27-item Conners’ Parent Rating Scales-Revised: Short Form (CPRS-R:S) and the 28-item Conners’ Teacher Rating Scales-Revised: Short Form (CTRS-R:S). Both forms have four different subscales: Cognitive problems/Inattention, Hyperactivity-Impulsivity, Oppositionality, and ADHD Index. All the items were rated on a 4-point Likert scale with 0 for not at all (never), 1 for just a little (occasionally), 2 for quite a bit (often), and 3 for very much (very often). These scales are reliable and valid instruments for measuring ADHD-related symptoms (6, 19, 70–72).

#### Quantity of Missing Data

We combined samples from two studies on ADHD. A total of 1220 youths completed CCPT assessments, of which 787 (64.5%) had SNAP-IV parent form, 575 (47.1%) had SNAP-IV teacher form, 995 (81.6%) had CPRS-R:S, and 729 (59.8%) had CTRS-R:S, and 462 (37.9%) had all four rating scales (see **Table 1**). Our goal is to use the CCPT data and the remaining complete scales to impute missing values for the incomplete scales.

**Table 1:** Data distribution for the ADHD and TD groups.

Number of Scales Completed	ADHD group(n = 799)		TD group(n = 421)	
	Boys(n = 689)	Girls(n = 110)	Boys(n = 343)	Girls(n = 78)
**Single scale**				
CPRS-R:S	42	8	20	1
CTRS-R:S	2	1	8	2
SNAP-IV-P	11	2	0	0
SNAP-IV-T	1	1	0	0
**Dual scales**				
CPRS-R:S, CTRS-R:S	112	18	54	20
CPRS-R:S, SNAP-IV-P	129	22	38	10
CPRS-R:S, SNAP-IV-T	0	0	0	0
CTRS-R:S, SNAP-IV-P	1	0	0	0
CTRS-R:S, SNAP-IV-T	9	0	6	0
SNAP-IV-P, SNAP-IV-T	42	8	0	0
**Triple scales**				
CPRS-R:S, CTRS-R:S, SNAP-IV-P	15	4	2	0
CPRS-R:S, CTRS-R:S, SNAP-IV-T	3	2	0	0
CPRS-R:S, SNAP-IV-P, SNAP-IV-T	26	4	2	1
CTRS-R:S, SNAP-IV-P, SNAP-IV-T	6	1	1	0
**Quad scales**				
CPRS-R:S, CTRS-R:S, SNAP-IV-P, SNAP-IV-T	222	33	170	37
**Completed scales with missing value detected**	68	6	42	7

Each element shows the number of participants who completed different scales included in this study. ADHD, attention-deficit/hyperactivity disorder; TD, typically developing; CPRS-R:S, the Chinese version of the Conners’ parent rating scales-revised: short form; CTRS-R:S, the Chinese version of the Conners’ teacher rating scales-revised: short form; SNAP-IV-P, the Chinese version of the Swanson, Nolan, and Pelham version IV scale, parent form; SNAP-IV-T, the Chinese version of the Swanson, Nolan, and Pelham version IV scale, teacher form.

#### Deep Neural Network for Missing Data Imputation

**Figure 1:**
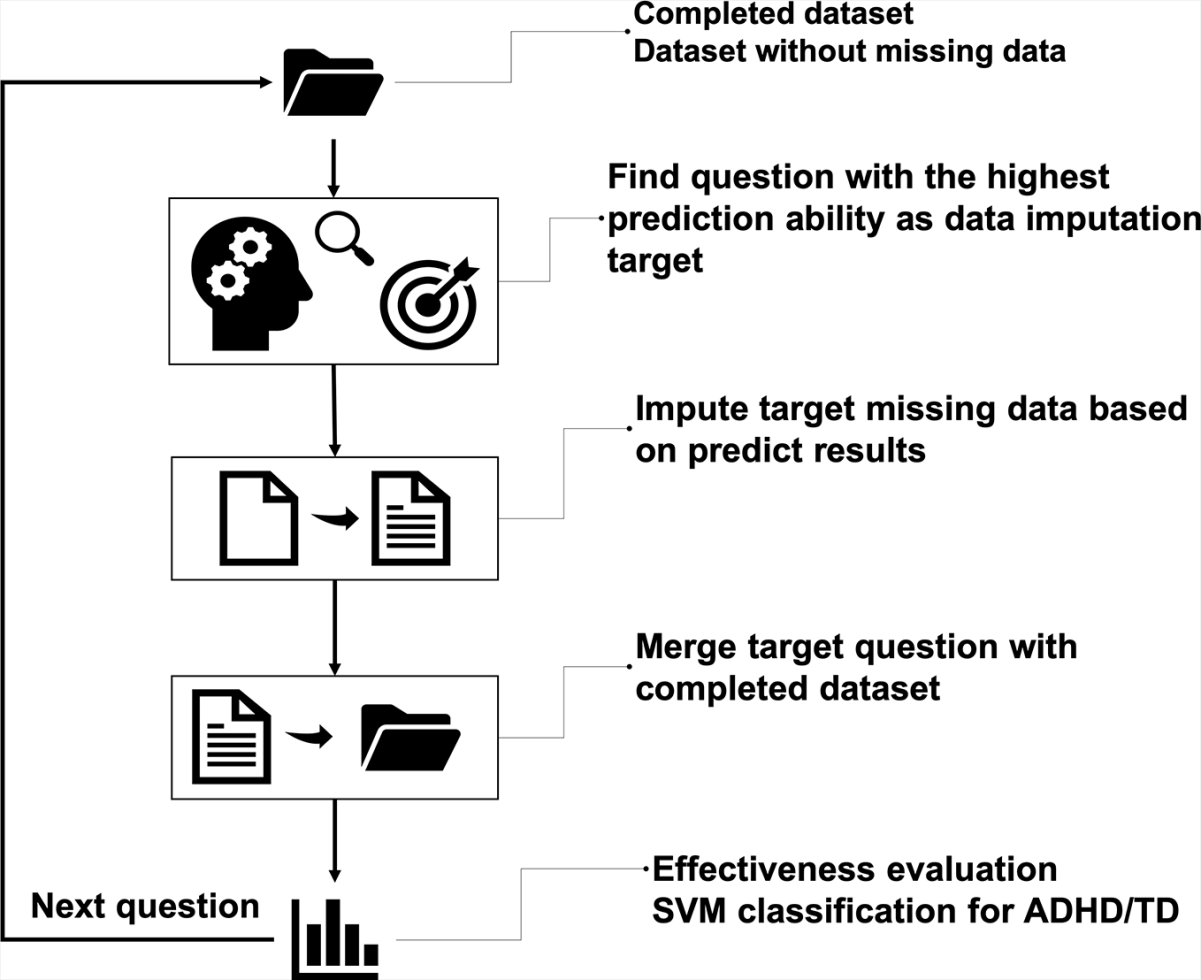
The overall flow of missing data imputation. The main idea is to iteratively select candidate questions for imputation and merge the imputed question into the complete dataset until every question had no missing data.

The interior architecture we used here is deep neural networks (DNN), which stacked modules that have multiple hidden layers and many neurons (87). It is also known as multi-layer perceptron (MLP), which is ANN mimicking human brains (88). DNN uses gradient descendent with backpropagation to train the algorithm, making the training process more efficient (89, 90). In this study, we designed an iteration framework to impute the ADHD data (see **Figure 1**). We used the 12 indices on the CCPT as the features of the initial training feature to start the imputation process. First, the DNN was used with all the questions of the four ADHD rating scales to identify the question with the highest accuracy to impute the missing values. Then this particular question was merged into the initial training set such that our training set now has one more feature to predict the next question. After these steps, the process moved back to the initial step and identified the next question with the highest predictability, iteratively.

**Figure 2:**
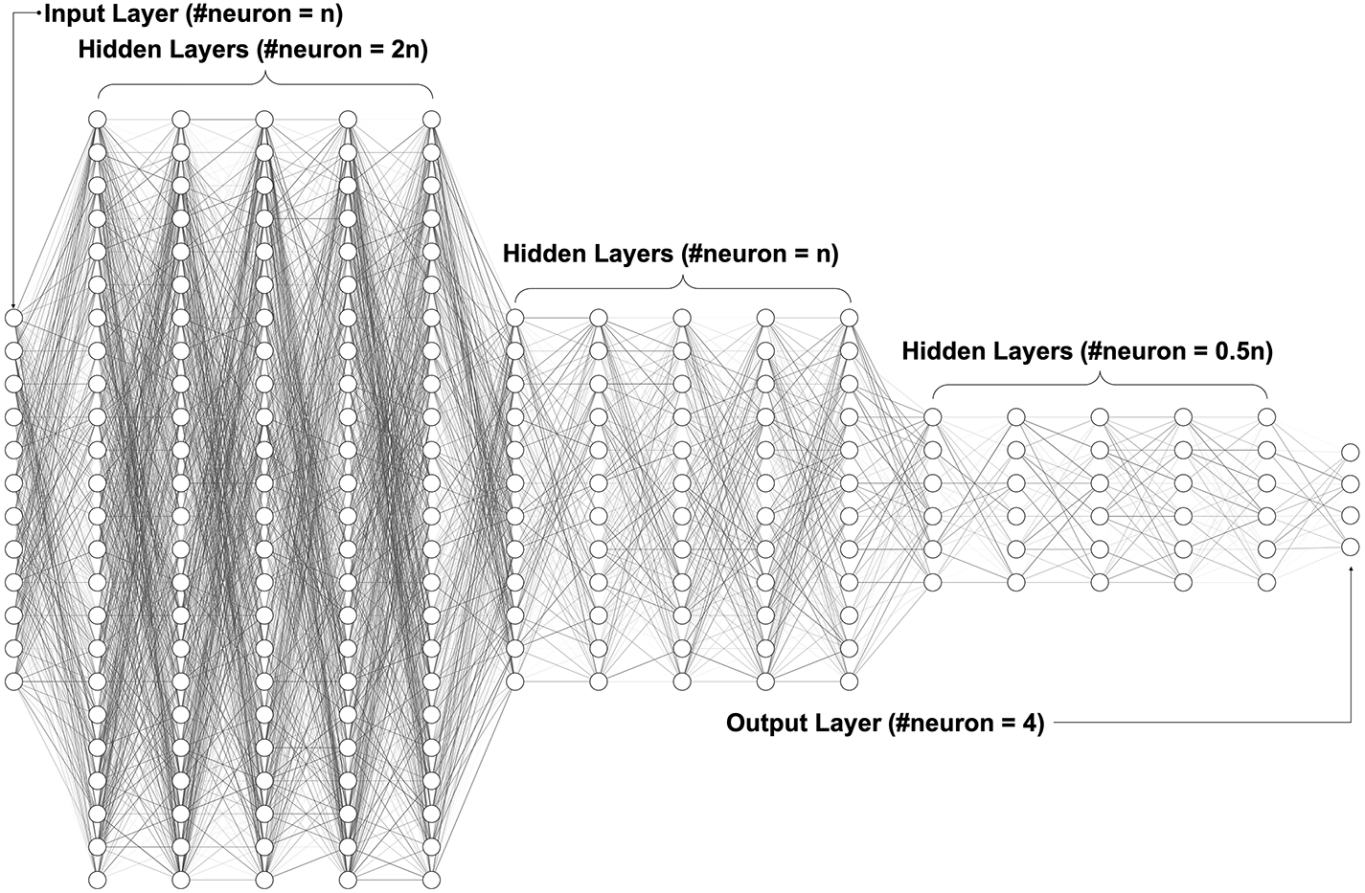
Deep neural network architecture. The fully connected neural network with one input layer, multiple hidden layers, and one output layer. The hidden layers use ReLU (Rectified Linear Unit) as the activation function, while the output layer uses Softmax to convert values to probabilities for the classification.

**Figure 2** shows our neural network architecture design, which included one input layer, 15 hidden layers, and one output layer. The number of neurons in the input layer started at 12 and increased by one with each iteration. The number of neurons in each hidden layer changed according to the number of input layer’s neurons. There was a total of 15 hidden layers divided into three groups: the beginning five hidden layers had twice the number of neurons of the input layer; the middle five hidden layers had the same number of neurons of the input layer; the last five hidden layers had half the number of neurons of the input layer. Since all scales are on a four-point Likert scale, we had four neurons in the output layer to represent the four possible scores. All layers’ activator, except for the output layer, was the Rectified Linear Unit (ReLU), which is one of the most common activators in deep learning (91), given its calculation speed, convergence speed and that it is gradient vanishing free. For the output layer, we chose the Softmax function, which converted values to probabilities for the four-point classification (92). To evaluate learning performance, we set up an SVM classification (93) to classify ADHD and TD after each iteration.

Deep learning has raised several concerns about hyper-parameters, which affect the speed and quality of the learning process (94, 95). One primary concern about deep learning is overfitting. To prevent this problem, we inserted dropout regularization in every layer (95). It will randomly abandon neurons after updating the weight of each layer. In addition, the iteration was optimized by adding early stopping and changing the batch size. The early stopping has a hyper-parameter called patience. If the training performance stopped improving after a certain number (defined as patience) of the pre-defined epoch (i.e., out of patience), training would stop. The patience of early stopping can significantly affect the whole process time, and batch size can affect model convergence speed; these methods not only can further prevent overfitting but also reduce unnecessary calculation (96). Specifically, we trained the algorithm using different combinations of parameters to find the best combination for our data. First, we used an early stopping function and picked patience on 10 and 100 epochs for this study. Larger epochs give the machine more steps to improve accuracy. Second, we set dropout rate at 20%, 25%, and 50% to evaluate overfitting (95). Lastly, we ran several different batch sizes to examine how batch size influenced deep learning algorithms (97–99). Batch Gradient Descent is where the batch size is equal to the size of the training set; batch size between 1 and the size of the training set is called Mini-Batch Gradient Descent (we used batch size=8 for the Mini-Batch). We also used a Stochastic Gradient Descent, where the batch size is one. The gradient and the neural network parameter updated after each batch sample. Because the Stochastic Gradient Descent (with batch size=1) needs lots of time to process, we only ran this with ten epochs for early stopping and 25% dropout rate.

#### Effectiveness Evaluation

We imputed missing data with the DNN analysis. After that, we conducted SVM classification (93) with the imputed data to distinguish between the ADHD and TD groups. SVM is a reliable machine learning classifier that has been used in many different clinical studies to classify disorders (100–103). By observing the classification score during every iteration, we found that the predictive power changed through our data imputation. After we finished missing data imputation, we used the imputation dataset and the reference dataset to run SVM classification with 10-fold cross-validation and then compared the prediction accuracy of the two datasets by using independent t-tests.

## Results

### Missing Data Imputation

#### Classification Accuracy Over Iterations

**Figure 3:**
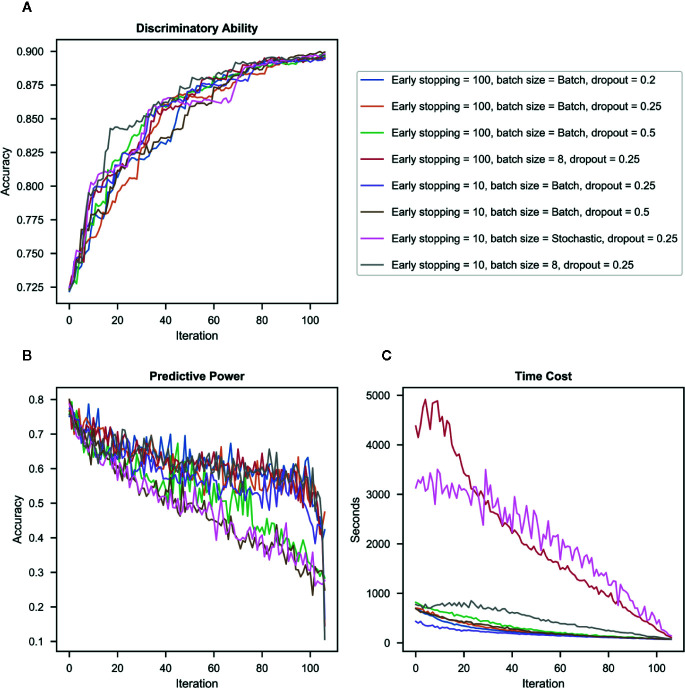
Results in different combinations of hyper-parameters. **(A)** Discriminatory ability – the accuracy of the imputed dataset in classifying ADHD vs. typically-developing (TD) controls for each iteration. **(B)** Predictive power – the accuracy of deep neural network (DNN) to impute candidate question’s missing values for each iteration. **(C)** Time cost – the processing time required for imputation for each iteration.

At the end of each iteration, we conducted SVM classification to classify ADHD and TD and recorded the classification accuracy. The classification accuracy increased from 72% to 90% from the first iteration to the last iteration. Despite some differences in classification accuracy in the middle of the whole iteration process, the accuracy of all the models (with different hyper-parameters) increased throughout iterations and achieved similar accuracy at the end of iterations (see **Figure 3A**).

#### Imputation Accuracy Over Iterations

**Figure 3B** presents the imputation accuracy after each iteration (when a question with the highest accuracy was identified, and its missing value was imputed and merged into the original training set) by different combinations of hyper-parameters. Results showed that accuracy decreased with iterations in all hyper-parameters. The dropout rate was the most influential contributor to the imputation accuracy, i.e., the model with a higher dropout rate had lower accuracy than those with a lower dropout rate in the same iteration. Batch size also influenced the imputation accuracy, i.e., the Stochastic approach has the lowest imputation accuracy even when used with a low dropout rate.

#### Time Cost

**Figure 3C** presents the processing time required for each iteration by different combinations of hyper-parameters. Results showed that as batch sizes decreased, the processing time increased. Batch mode was the most time-efficient.

### Effectiveness Evaluation

To evaluate the success of imputation, we used SVM classification to examine the ability of the imputed dataset (n =758, 62.1%), estimated with different combinations of hyper-parameters, to classify between the ADHD vs. TD groups. We then conducted independent t-tests to compare the classification accuracy of each of these datasets to that of the reference dataset i.e., the original dataset for which all the four scales were complete (n=462, 37.9%). Results showed that different imputed datasets shared similar mean accuracy (0.89 to 0.90), which was not significantly different from the reference dataset (accuracy = 0.89) (see **Table 2**). Among the imputation datasets, we did not observe much difference in accuracy between datasets imputed with different dropout rates and batch sizes, suggesting that these factors did not influence the predictive power of the imputed data to distinguish ADHD from TD.

**Table 2:** Classification accuracy of attention-deficit/hyperactivity disorder (ADHD) vs. typically developing (TD) controls in models with different combinations of hyper-parameters (early stopping, batch size, and dropout rate).

Hyper-parameter	ATCACC	*p*-value
The original group with the completed dataset	0.8931 ± 0.0215	
ES=100, BS=Batch, D=0.25	0.8859 ± 0.0142	0.415
ES=100, BS=Batch, D=0.2	0.8987 ± 0.0139	0.523
ES=100, BS=Batch, D=0.5	0.8954 ± 0.0164	0.801
ES=100, BS=8, D=0.25	0.8918 ± 0.0113	0.875
ES=10, BS=Batch, D=0.25	0.8885 ± 0.0118	0.585
ES=10, BS=Batch, D=0.5	0.899 ± 0.0121	0.484
ES=10, BS=8, D=0.25	0.8921 ± 0.0109	0.906
ES=10, BS=Stochastic, D=0.25	0.9046 ± 0.0159	0.216

Results presented as means ± standard deviations. Independent t-tests were used to compare the classification accuracy between the imputed and reference datasets. ATCACC, ADHD/TD classification accuracy; ES, earlystopping patience; BS, batch size; D, dropout rate.

### Imputation Order of Questions

All the items (107 in total) in the four scales were categorized into three groups by the imputation order (see **Supplementary Table 1**): (1) Top group: items that had high *discrimination* accuracy and were picked up by the machine early (35 items), (2) Bottom group: items that had low accuracy and did not become a target for imputation until other items with higher imputed accuracy were picked (35 items), and (3) Intermediate group (37 items).

Top group: Both parent and teacher reports on questions assessing oppositional behaviors such as “spiteful or vindictive” demonstrated the highest ability to discriminating ADHD from TD. Moreover, questions assessing hyperactive-impulsive symptoms, such as “leaves seat,” “runs about or restless,” and “impatient,” were also included in this group. Some questions reported only by the teachers were also in this group e.g., “argues with adults,” “actively defies or refuses adult requests or rules,” “is angry and resentful” and “avoids, expresses reluctance about, or has difficulties engaging in tasks that require sustained mental effort (such as schoolwork or homework).”

Intermediate group: Most questions included in this group were about inattention e.g., “cannot pay attention,” “fails to finish work,” “disorganized,” “cannot concentrate,” “distractible,” “not reading up to par,” “poor in arithmetic,” and “forgets things he or she has already learned.” Several impulsive questions, such as “intrudes on others” and “blames others for his or her mistakes,” were also included in this group.

Bottom group: Both parent and teacher reports on questions such as “talks excessively,” “only pays attention to things he/she is interested in,” “loses things,” and “makes careless mistakes” did not have high discriminatory accuracy. In contrast to the high accuracy of teacher reports of several oppositional behaviors, parent reports on these questions fell into this group.

## Discussion

The current study, to the best of our knowledge, is the first work using deep learning to impute missing data in ADHD-related rating scales. Three main findings emerged. First, missing data can be imputed using deep learning with high accuracy and that the imputed dataset had a similar, high discriminatory ability to distinguish between the ADHD and TD groups compared to the complete original dataset. Second, our approach generated an imputation order of questions, demonstrating that teacher-reported oppositional symptoms and both teacher- and parent-reported hyperactivity-impulsivity symptoms were highly discriminating symptom clusters to distinguish between the ADHD and TD groups. Third, changing hyper-parameters in deep learning affects the analysis processes and results i.e., deep learning performance is sensitive to hyper-parameters. This study focuses on manipulating batch size, dropout rate, and early stopping. By changing these hyper-parameters, we partially verified some previous findings suggesting that batch size and early stopping have a large effect on processing time and that the dropout rate is the most relevant hyper-parameter for predictive power (97–99, 104).

With our approach, the missing data can be imputed using deep learning, and the imputed dataset has the same high discriminatory ability as the original complete dataset (i.e., the reference dataset). Our results provide strong evidence to support that our imputation not only generated the dataset without missing values but also kept the imputed and reference datasets consistent. Of note, one novelty of this deep learning approach is that we let the machine impute missing data for the whole sample combining the ADHD and TD groups. Previous studies typically only focus on one group imputation at a time, given concerns about biasing the subsequent analyses when combining the case and the control groups during the imputation process (66, 105, 106). For example, combining the two groups may decrease differences and increase the similarity between the case and control groups in terms of the distribution of features. In contrast, imputing missing value separately by groups may introduce bias to the distribution of features, enlarge the group difference (107), and lead to increasing discriminatory ability after imputation. As a result, if new data are added later on, the discriminatory ability might drop because the feature distribution of the imputed dataset is not representative of the original dataset. Our method represents a novel solution to impute missing data while maintaining the discriminatory ability of the imputed dataset to distinguish between the ADHD and non-ADHD groups. This strategy can also ensure that when new data are available at a later time, they can be readily added to and mixed with the imputed data.

This study imputed 45,229 missing values. Each question has a different amount of missing data. About 200 participants had one or two missing questions, and more than 600 participants had missing data in some questions of the four scales. Our result showed that there is no relation between the order of missing data imputation and the amount of missing data in the questions. Even though the original dataset has about 60% missing items across the four scales, our finding showed that the machine could still learn from this dataset. What matters more is to have questions that are highly discriminative between ADHD and non-ADHD. If a question lacks discriminative ability but has minimal amount of missing data, our algorithm would select another question that has higher discriminative ability because the machine would always pick the best feature in each iteration. Our classification accuracy between the ADHD and TD groups increased rapidly at the beginning of the iteration after the missing values for the highly-discriminative questions were imputed. At the end of the iteration, the imputed dataset had the same classification accuracy and distribution compared to the original complete dataset (reference dataset). The most critical issue of data imputation is the bias that it may introduce, ultimately affecting the inferences that can be drawn from the analysis conducted with the imputed dataset. We also compared our classification accuracy with other imputation methods (i.e., interpolate imputation, mean imputation, and multiple imputation). Results indicated that deep learning approach have higher accuracy than traditional statistical imputation methods (see **Supplementary Table 2**). Our results suggest that deep learning can be a robust and reliable method for handling missing data to generate an imputed dataset resembling the reference dataset and that subsequent analyses conducted with the imputed data showed consistent results with those from the reference dataset.

Imputation order is another important finding of this study. The high-order questions, relative to the low-order ones, are assumed to have higher discriminant validity to differentiate children with ADHD from those without ADHD. Consistent with our hypotheses, our results showed that most of the hyperactivity-impulsivity questions, from both teacher and parent reports, fell into the high-order group. Hyperactive-impulsive behaviors, the “externalizing” features of ADHD, are easily observed in various settings. For example, behavioral descriptions such as “leaves the seat,” “fidgety,” and “runs about or climbs” provide specific behaviors for parents and teachers to rate the child in a precise manner. This may be why these hyperactivity-impulsivity questions have high discriminatory validity. However, when hyperactive questions were worded metaphorically such as “restless in the squirmy sense,” “acts as if driven by a motor,” and “talks excessively,” parents and teachers seemed to have a hard time providing valid ratings as indexed by the low discriminatory accuracy of these questions.

Interestingly, we found that almost all oppositional questions from the teacher report were categorized into the high-order group, whereas oppositional questions from the parent report were in the low-order group (67). That is, according to these internationally well-known standardized scales used in our ADHD studies, teacher reports of oppositional symptoms had better discriminant validity in distinguishing ADHD from non-ADHD. One possible explanation is that the classroom teachers, in general, spend more time with the students than parents do and are more likely to observe oppositional symptoms of the index children against a group norm of the same-age peers (6). Given the high co-occurrence between ADHD and ODD symptoms, this may be why teachers’ observations of children’s ODD symptoms had better discriminant validity in distinguishing ADHD from non-ADHD.

Our goal is to impute the missing data of the scales; however, there are some of items in the scales designed for screening ODD symptoms, which are not ADHD symptoms but highly co-occurring with ADHD (CPRS-R:S: 2,6,11,16,20,24; CTRS-R:S: 2,6,10,15,20; SNAP-IV-P: 19-26; SNAP-IV-T: 19-21,23-26,29). We also conducted analyses without the ODD symptoms (see **Supplementary Figure 1**). The ADHD/TD classification accuracy showed no difference between our original results with ODD symptoms included and the results with ODD symptoms excluded (see **Supplementary Table 3**). Comparing the imputation orders with the results with ODD symptoms (see **Supplementary Table 1** and **Supplementary Table 4**) and those without ODD symptoms (see **Supplementary Table 3** and **Supplementary Table 5**), the imputation order of the same items across the two sets of analyses did not change. Our findings of no differences in the imputation orders of other symptoms rather than ODD symptoms between the two analyses suggest that removing ODD items did not affect machine classification, and removing some of the items from the scales did not affect the machine’s ability to learn.

Of note, both parent and teacher reports of inattention questions showed low discriminatory accuracy (108). One possible explanation, as described in the introduction, is that inattention is more difficult to observe than other externalizing symptoms. In addition, every child forgets things or is careless occasionally. Therefore, these types of behaviors may be viewed as normative by parents and teachers (109, 110). However, one exception is that “avoids, expresses reluctance about, or has difficulties engaging in tasks that require sustained mental effort (such as schoolwork or homework)” reported by the teachers on the SNAP-IV was included in the high order group. This suggests that teachers’ evaluation and observation of an index student’s schoolwork and submitted homework as compared to same-age students at the school can distinguish ADHD from non-ADHD.

Our results also showed that changing hyper-parameters (e.g., batch size, dropout rate) in deep learning may affect the performance of the algorithm. We processed with different batch sizes (Batch [size=training set], Mini-batch [size=8], and Stochastic [size=1]) to evaluate the outcomes of the discriminatory accuracy, a hot topic in the deep learning field (97–99, 104). We found that across various sizes of the batch, the discriminative ability to separate ADHD from TD reached the same accuracy after missing data imputation. That is, decreasing batch sizes did not improve the accuracy further, but took much longer to process with deep learning. Batch mode is the most time-efficient. Although Mini-batch requires less memory during processing, hardware advances today have afforded us the memory required for deep learning, making Mini-batch not advantageous over other batch sizes in this aspect. We also processed with Stochastic (batch size=1) to verify the idea of online learning performance (111). The outcome of this showed that the performance was not on par with the mini-batch mode during every iteration of the imputation process, and it took more time to converge than the batch mode. In summary, batch mode allows the machine to compute the gradient over the entire dataset, leveraging an abundant amount of information to find a proper solution more efficiently.

There are several methodological limitations in our study. First, due to the architecture based on the deep learning approach, the larger the size of data is, the more thoroughly the machine can learn. Although our sample size is more than 1,000, this may not be sufficiently large for deep learning. However, for the clinical data with excellent quality and internal validity collected from a single site, our sample size is rather large. Second, our imputation approach combined the ADHD and TD groups, resulting in the machine having to learn more varying values in each feature with a limited sample size. Hence, future research with larger sample sizes is also warranted in this aspect. Third, although our imputed dataset had the same accuracy as the original complete data in classifying the ADHD and TD groups, there is no guarantee that the imputed values are “accurate.” Indeed, our results showed that the predictive power (i.e., the accuracy in predicting the rating scale scores) decreased over time with iterations. This suggests that the machine performed poorly for some items, especially when imputing missing scores for the bottom third of the questions. Fourth, although we used the clinician’s diagnosis (which is based on observations and interviews of the patient as well as interviews with the parent/caregiver) as the outcome to evaluate the effectiveness of imputation and parent questionnaires as part of the features for imputation, the shared variance from parent reports either through answering questions from the clinician or as self-response to the questionnaires is a methodological limitation and potential confounder. Future investigation is warranted to add more features from other informants, e.g., participant’s self-reports, peers reports, or other objective measures. Lastly, although we designed flexible neuron size of each hidden layer to adapt to the number of increasing neurons needed for each input layer, as the number of hidden layers is static, it might lead the last few iterations of imputed output layer to over converge than expected.

## Conclusions

We present a novel approach to impute missing data in ADHD rating scales based on deep learning using participants’ neuropsychological data and ADHD-related behaviors assessed with four scales reported by parents and teachers. Our deep learning approach can impute missing data with both the case and control groups together in the dataset. Our findings provide evidence that our deep learning approach can impute missing data with high accuracy in an aggregated dataset from multiple samples and thus can increase the size of the dataset while maintaining the characteristics and representativeness of the data’s original distribution.

## Data Availability Statement

The raw data supporting the conclusions of this article will be made available by the corresponding author only if this request is approved by the Research Ethics Committee of National Taiwan University Hospital, Taipei, Taiwan, according to the current regulation of patient protection in Taiwan.

The datasets used in the analysis were collected using the grants support to the corresponding author (SS-FG) mentioned in the acknowledgments section. The data are available upon request. The studies were preregistered at ClinicalTrials.gov number: NCT00529906 (NSC96-2628-B-002-069-MY3), NCT00916786 (NSC98-2314-B-002-051-MY3), and NCT00417781 (NHRI-EX94~98-9407PC).

## Ethics Statement

The studies involving human participants were reviewed and approved by the Research Ethics Committee of National Taiwan University Hospital, Taipei, Taiwan (Approval numbers: 200612114R, 200812153M, 9361700470; ClinicalTrials.gov number: NCT00529906, NCT00916786, NCT00417781). Written informed consent to participate in this study was provided by the participants’ legal guardian/next of kin.

## Author Contributions

Author contributions included conception and study design (C-YC and SS-FG), data collection and acquisition (SS-FG), statistical analysis (C-YC, C-FC, C-HC), interpretation of results (C-YC, W-LT, C-FC, C-HC and SS-FG), drafting the manuscript work (C-YC, W-LT) and revising it critically for important intellectual content (C-YC, W-LT, and SS-FG), and approval of the final version to be published and agreement to be accountable for the integrity and accuracy of all aspects of the work (all authors).

## Conflict of Interest

The authors declare that the research was conducted in the absence of any commercial or financial relationships that could be construed as a potential conflict of interest.

## References

[1] American Psychiatric Association Diagnostic and statistical manual of mental disorders, Fifth Edition American Psychiatric Association, Arlinton, VA (2013).

[2] Gau SS Chong M-Y Chen TH Cheng AT A 3-year panel study of mental disorders among adolescents in Taiwan. Am J Psychiatry (2005) 162:1344–50. 10.1176/appi.ajp.162.7.1344 15994718

[3] Danielson ML Bitsko RH Ghandour RM Holbrook JR Kogan MD Blumberg SJ Prevalence of parent-reported ADHD diagnosis and associated treatment among US children and adolescents, 2016. J Clin Child Adolesc Psychol (2018) 47:199–212. 10.1080/15374416.2017.1417860 29363986 PMC5834391

[4] Chen Y-Y Chen Y-L Gau SS-F Attention-deficit hyperactivity disorder and suicidality: The mediating effects of psychiatric comorbidities and family function. J Affect Disord (2019) 242:96–104. 10.1016/j.jad.2018.08.023 30173064

[5] Wu S-Y Gau SS-F Correlates for academic performance and school functioning among youths with and without persistent attention-deficit/hyperactivity disorder. Res Dev Disabil (2013) 34:505–15. 10.1016/j.ridd.2012.09.004 23063730

[6] Kawabata Y Tseng W-L Gau SS-F Symptoms of attention-deficit/hyperactivity disorder and social and school adjustment: The moderating roles of age and parenting. J Abnormal Child Psychol (2012) 40:177–88. 10.1007/s10802-011-9556-9 21858455

[7] Bauermeister JJ Barkley RA Bauermeister JA Martínez JV McBurnett K Validity of the sluggish cognitive tempo, inattention, and hyperactivity symptom dimensions: Neuropsychological and psychosocial correlates. J Abnormal Child Psychol (2012) 40:683–97. 10.1007/s10802-011-9602-7 22179974

[8] Liu T-L Guo N-W Hsiao RC Hu H-F Yen C-F Relationships of bullying involvement with intelligence, attention, and executive function in children and adolescents with attention-deficit/hyperactivity disorder. Res Dev Disabil (2017) 70:59–66. 10.1016/j.ridd.2017.08.004 28898705

[9] Chang L-R Chiu Y-N Wu Y-Y Gau SS-F Father’s parenting and father–child relationship among children and adolescents with attention-deficit/hyperactivity disorder. Compr Psychiatry (2013) 54:128–40. 10.1016/j.comppsych.2012.07.008 22985803

[10] Lin H-Y Tseng W-Y Lai M-C Chang Y-T Gau S-F Shared atypical brain anatomy and intrinsic functional architecture in male youth with autism spectrum disorder and their unaffected brothers. Psychol Med (2017) 47:639–54. 10.1017/S0033291716002695 27825394

[11] Gau SS-F Ni H-C Shang C-Y Soong W-T Wu Y-Y Lin L-Y Psychiatric comorbidity among children and adolescents with and without persistent attention-deficit hyperactivity disorder. Aust New Z J Psychiatry (2010) 44:135–43. 10.3109/00048670903282733 20113302

[12] Han GT Chen Y-L Tsai F-J Gau SS-F Temporal and reciprocal relations between ADHD symptoms and emotional problems in school-age children. J Attention Disord (2018) 24:1032–44. 10.1177/1087054718787891 PMC6675667 30066607

[13] Lin Y-J Gau SS-F Differential neuropsychological functioning between adolescents with attention-deficit/hyperactivity disorder with and without conduct disorder. J Formosan Med Assoc (2017) 116:946–55. 10.1016/j.jfma.2017.02.009 28292624

[14] Faraone S Asherson P Banaschewski T Biederman J Buitelaar J Ramos-Quiroga J Attention-deficit/hyperactivity disorder. Nat Rev: Dis Primers (2015) 1:15020. 10.1038/nrdp.2015.20 27189265

[15] Lin Y-J Yang L-K Gau SS-F Psychiatric comorbidities of adults with early-and late-onset attention-deficit/hyperactivity disorder. Aust New Z J Psychiatry (2016) 50:548–56. 10.1177/0004867415609423 26460330

[16] Lin Y-J Gau SS-F Comparison of Neuropsychological Functioning Between Adults With Early-and Late-Onset DSM-5 ADHD. J Attention Disord (2017) 24:29–40. 10.1177/1087054717730609 28895460

[17] Lin Y-J Gau SS-F Developmental changes of neuropsychological functioning in individuals with and without childhood ADHD from early adolescence to young adulthood: a 7-year follow-up study. Psychol Med (2019) 49:940–51. 10.1017/S0033291718001599 29941053

[18] Lin Y-J Lo K-W Yang L-K Gau SS-F Validation of DSM-5 age-of-onset criterion of attention deficit/hyperactivity disorder (ADHD) in adults: Comparison of life quality, functional impairment, and family function. Res Dev Disabil (2015) 47:48–60. 10.1016/j.ridd.2015.07.026 26318976

[19] Gau SS-F Soong W-T Chiu Y-N Tsai W-C Psychometric properties of the Chinese version of the Conners’ parent and teacher rating scales-revised: Short form. J Attention Disord (2006) 9:648–59. 10.1177/1087054705284241 16648232

[20] Chen C-H Chen H-I Chien W-H Li L-H Wu Y-Y Chiu Y-N High resolution analysis of rare copy number variants in patients with autism spectrum disorder from Taiwan. Sci Rep (2017) 7:11919. 10.1038/s41598-017-12081-4 28931914 PMC5607249

[21] Gau SS-F Lin C-H Hu F-C Shang C-Y Swanson JM Liu Y-C Psychometric properties of the Chinese version of the Swanson, Nolan, and Pelham, version IV scale-Teacher Form. J Pediatr Psychol (2008) 34:850–61. 10.1093/jpepsy/jsn133 19074488

[22] Gau SSF Shang CY Liu SK Lin CH Swanson JM Liu YC Psychometric properties of the Chinese version of the Swanson, Nolan, and Pelham, version IV scale–parent form. Int J Methods Psychiatr Res (2008) 17:35–44. 10.1002/mpr.237 18286459 PMC6878250

[23] Gau SS Huang Y-S Soong W-T Chou M-C Chou W-J Shang C-Y A randomized, double-blind, placebo-controlled clinical trial on once-daily atomoxetine hydrochloride in Taiwanese children and adolescents with attention-deficit/hyperactivity disorder. J Child Adolesc Psychopharmacol (2007) 17:447–60. 10.1089/cap.2006.0091 17822340

[24] Ni H-C Hwang Gu S-L Lin H-Y Lin Y-J Yang L-K Huang H-C Atomoxetine could improve intra-individual variability in drug-naïve adults with attention-deficit/hyperactivity disorder comparably with methylphenidate: a head-to-head randomized clinical trial. J Psychopharmacol (2016) 30:459–67. 10.1177/0269881116632377 26905919

[25] Shang C Yan C Lin H Tseng W Castellanos F Gau S Differential effects of methylphenidate and atomoxetine on intrinsic brain activity in children with attention deficit hyperactivity disorder. psychol Med (2016) 46:3173–85. 10.1017/S0033291716001938 27574878

[26] Gau SS Huang YS Soong WT Chou MC Chou WJ Shang CY A randomized, double-blind, placebo-controlled clinical trial on once-daily atomoxetine in Taiwanese children and adolescents with attention-deficit/hyperactivity disorder. J Child Adolesc Psychopharmacol (2007) 17:447–60. 10.1089/cap.2006.0091 17822340

[27] Ni HC Hwang Gu SL Lin HY Lin YJ Yang LK Huang HC Atomoxetine could improve intra-individual variability in drug-naive adults with attention-deficit/hyperactivity disorder comparably with methylphenidate: A head-to-head randomized clinical trial. J Psychopharmacol (2016) 30:459–67. 10.1177/0269881116632377 26905919

[28] Liu Y-C Norm of the Chinese version of the Swanson, Nolan, and Pelham, version IV scale for ADHD. Taiwan J Psychiatry (2006) 20:209–304. 10.29478/TJP.200612.0006

[29] Chien Y-L Chou M-C Chiu Y-N Chou W-J Wu Y-Y Tsai W-C ADHD-related symptoms and attention profiles in the unaffected siblings of probands with autism spectrum disorder: focus on the subtypes of autism and Asperger’s disorder. Mol Autism (2017) 8:37. 10.1186/s13229-017-0153-9 28770037 PMC5526322

[30] Miranda MC Barbosa T Muszkat M Rodrigues CC Sinnes EG Coelho LFS Performance patterns in Conners’ CPT among children with attention deficit hyperactivity disorder and dyslexia. Arquivos Neuro-psiquiatr (2012) 70:91–6. 10.1590/S0004-282X2012000200004 22311211

[31] Berger I Slobodin O Cassuto H Usefulness and validity of continuous performance tests in the diagnosis of attention-deficit hyperactivity disorder children. Arch Clin Neuropsychol (2017) 32:81–93. 10.1093/arclin/acw101 28122767

[32] Hwang-Gu S-L Lin H-Y Chen Y-C Y.-h. Tseng W-Y Chou M-C Chou W-J Symptoms of ADHD Affect Intrasubject Variability in Youths with Autism Spectrum Disorder: An Ex-Gaussian Analysis. J Clin Child Adolesc Psychol (2019) 48:455–68. 10.1080/15374416.2018.1452151 29847154

[33] Arble E Kuentzel J Barnett D Convergent validity of the Integrated Visual and Auditory Continuous Performance Test (IVA+ Plus): associations with working memory, processing speed, and behavioral ratings. Arch Clin Neuropsychol (2014) 29:300–12. 10.1093/arclin/acu006 24687587

[34] Su C-T Gau S-F Soong W-T Sheu C-F Chiu Y-N Tsai W-C Examination of the Psychometric Properties of the Conners. Taiwanese J Psychiatry (2009) 23:146–58. 10.29478/TJP.200906.0007

[35] Narad ME Garner AA Peugh JL Tamm L Antonini TN Kingery KM Parent–teacher agreement on ADHD symptoms across development. psychol Assess (2015) 27:239–48. 10.1037/a0037864 PMC4495952 25222436

[36] Reh V Schmidt M Lam L Schimmelmann BG Hebebrand J Rief W Behavioral assessment of core ADHD symptoms using the QbTest. J Attention Disord (2015) 19:1034–45. 10.1177/1087054712472981 23382579

[37] Chen Y-C Hwang-Gu S-L Ni H-C Liang SH-Y Lin H-Y Lin C-F Relationship between parenting stress and informant discrepancies on symptoms of ADHD/ODD and internalizing behaviors in preschool children. PloS One (2017) 12:e0183467. 10.1371/journal.pone.0183467 29016602 PMC5634535

[38] Chen Y-L Chen WJ Lin K-C Shen L-J Gau SS-F Prevalence of DSM-5 mental disorders in a nationally representative sample of children in Taiwan: methodology and main findings. Epidemiol Psychiatr Sci (2019) 29:e15. 10.1017/S2045796018000793 30696515 PMC8061245

[39] Schafer JL Graham JW Missing data: our view of the state of the art. psychol Methods (2002) 7:147–77. 10.1037/1082-989X.7.2.147 12090408

[40] McKnight PE McKnight KM Sidani S Figueredo AJ Missing data: A gentle introduction. Guilford Press (2007).

[41] Radloff LS The CES-D scale: A self-report depression scale for research in the general population. Appl Psychol Measurement (1977) 1:385–401. 10.1177/014662167700100306

[42] de Vet HC Terwee CB Mokkink LB Knol DL Measurement in medicine: a practical guide. Cambridge University Press (2011).

[43] Lyons RA Perry IM B.N. LITTLEPAGE Evidence for the validity of the Short-form 36 Questionnaire (SF-36) in an elderly population. Age Ageing (1994) 23:182–4. 10.1093/ageing/23.3.182 8085500

[44] Burton A Altman D Missing covariate data within cancer prognostic studies: a review of current reporting and proposed guidelines. Br J Cancer (2004) 91:4–8. 10.1038/sj.bjc.6601907 15188004 PMC2364743

[45] Eekhout I de Boer RM Twisk JW de Vet HC Heymans MW Missing data: a systematic review of how they are reported and handled. Epidemiology (2012) 23:729–32. 10.1097/EDE.0b013e3182576cdb 22584299

[46] Wood AM White IR Thompson SG Are missing outcome data adequately handled? A Rev Published Randomized Controlled Trials Major Med J Clin Trials (2004) 1:368–76. 10.1191/1740774504cn032oa 16279275

[47] Pigott TD A review of methods for missing data. Educ Res Eval (2001) 7:353–83. 10.1076/edre.7.4.353.8937

[48] Enders CK Applied missing data analysis. Guilford press (2010).

[49] Little RJ Rubin DB Statistical analysis with missing data. Vol. 793 John Wiley & Sons (2019).

[50] Hernán MA Robins JM Estimating causal effects from epidemiological data. J Epidemiol Community Health (2006) 60:578–86. 10.1136/jech.2004.029496 PMC2652882 16790829

[51] Rubin DB Multiple imputation for nonresponse in surveys. Vol. 81 John Wiley & Sons (2004).

[52] Huisman M Item nonresponse: Occurrence, causes, and imputation of missing answers to test items. Leiden: DSWO Press (1999).

[53] van Buuren S Item imputation without specifying scale structure. Methodology (2010) 6:31–6. 10.1027/1614-2241/a000004

[54] Baraldi AN Enders CK An introduction to modern missing data analyses. J School Psychol (2010) 48:5–37. 10.1016/j.jsp.2009.10.001 20006986

[55] Jerez JM Molina I García-Laencina PJ Alba E Ribelles N Martín M Missing data imputation using statistical and machine learning methods in a real breast cancer problem. Artif Intell Med (2010) 50:105–15. 10.1016/j.artmed.2010.05.002 20638252

[56] Fukushima K Miyake S Ito T Neocognitron: A neural network model for a mechanism of visual pattern recognition. IEEE Trans Syst Man Cybernet (1983) SMC-13:826–34. 10.1109/TSMC.1983.6313076

[57] Hinton GE Salakhutdinov RR Reducing the dimensionality of data with neural networks. science (2006) 313:504–7. 10.1126/science.1127647 16873662

[58] Durstewitz D Koppe G Meyer-Lindenberg A Deep neural networks in psychiatry. Mol Psychiatry (2019) 24:1583–98. 10.1038/s41380-019-0365-9 30770893

[59] Menke A Precision pharmacotherapy: psychiatry’s future direction in preventing, diagnosing, and treating mental disorders. Pharmacogenom Personalized Med (2018) 11:211–22. 10.2147/PGPM.S146110 PMC6250105 30510440

[60] Kuang D Guo X An X Zhao Y He L Discrimination of ADHD based on fMRI data with deep belief network. Intelligent Computing in Bioinformatics. ICIC 2014 Lecture Notes in Computer Science, vol 8590 Springer, Cham (2014), p. 225–32. 10.1007/978-3-319-09330-7_27

[61] Zou L Zheng J Miao C Mckeown MJ Wang ZJ 3D CNN based automatic diagnosis of attention deficit hyperactivity disorder using functional and structural MRI. IEEE Access (2017) 5:23626–36. 10.1109/ACCESS.2017.2762703

[62] Choi H-S Choe JY Kim H Han JW Chi YK Kim K Deep learning based low-cost high-accuracy diagnostic framework for dementia using comprehensive neuropsychological assessment profiles. BMC Geriatr (2018) 18:234. 10.1186/s12877-018-0915-z 30285646 PMC6171238

[63] Song Y Gao S Tan W Qiu Z Zhou H Zhao Y Multiple Machine Learnings Revealed Similar Predictive Accuracy for Prognosis of PNETs from the Surveillance, Epidemiology, and End Result Database. J Cancer (2018) 9:3971–8. 10.7150/jca.26649 PMC6218767 30410601

[64] Thung K-H Yap P-T Shen D Multi-stage diagnosis of Alzheimer’s disease with incomplete multimodal data via multi-task deep learning, Deep Learning in Medical Image Analysis and Multimodal Learning for Clinical Decision Support. DLMIA 2017, ML-CDS 2017. Lecture Notes in Computer Science, vol 10553. Springer, Cham. (2017), p. 160–8. 10.1007/978-3-319-67558-9_19 PMC5666687 29104963

[65] Qiu YL Zheng H Gevaert O A deep learning framework for imputing missing values in genomic data. bioRxiv [Preprint] (2018). Available at: https://www.biorxiv.org/content/10.1101/406066v2 (Accessed September 03, 2018). 10.1101/406066

[66] Beaulieu-Jones BK Moore JH Missing data imputation in the electronic health record using deeply learned autoencoders, Pacific Symposium on Biocomputing 2017 (2017), p. 207–18. 10.1142/9789813207813_0021 PMC5144587 27896976

[67] Hall CL Guo B Valentine AZ Groom MJ Daley D Sayal K The validity of the SNAP-IV in children displaying ADHD symptoms. Assessment (2019). 10.1177/1073191119842255 30991820

[68] Wechsler D WISC-III: Wechsler intelligence scale for children. Psychological Corporation (1991).

[69] Chiang M Gau SS Validation of attention-deficit–hyperactivity disorder subtypes among Taiwanese children using neuropsychological functioning. Aust New Z J Psychiatry (2008) 42:526–35. 10.1080/00048670802050603 18465380

[70] Hwang-Gu S-L Gau SS-F Interval timing deficits assessed by time reproduction dual tasks as cognitive endophenotypes for attention-deficit/hyperactivity disorder. PloS One (2015) 10:e0127157. 10.1371/journal.pone.0127157 25992899 PMC4436371

[71] Chiang HL Gau SSF Ni HC Chiu YN Shang CY Wu YY Association between symptoms and subtypes of attention-deficit hyperactivity disorder and sleep problems/disorders. J Sleep Res (2010) 19:535–45. 10.1111/j.1365-2869.2010.00832.x 20408926

[72] Tseng W-L Kawabata Y Gau SS-F Social adjustment among Taiwanese children with symptoms of ADHD, ODD, and ADHD comorbid with ODD. Child Psychiatry Hum Dev (2011) 42:134–51. 10.1007/s10578-010-0204-3 20886286

[73] Tsai C-J Chen Y-L Lin H-Y Gau SS-F One-year trajectory analysis for ADHD symptoms and its associated factors in community-based children and adolescents in Taiwan. Child Adolesc Psychiatry Ment Health (2017) 11:28. 10.1186/s13034-017-0165-4 28580012 PMC5452532

[74] Tai Y-M Gau SS-F Depression and quality of life mediating the association between attention deficit/hyperactivity disorder and suicidality in military recruits. Military Med (2017) 182:e1912–9. 10.7205/MILMED-D-16-00394 29087861

[75] Chiang HL Kao WC Chou MC Chou WJ Chiu YN Wu YY School dysfunction in youth with autistic spectrum disorder in Taiwan: The effect of subtype and ADHD. Autism Res (2018) 11:857–69. 10.1002/aur.1923 29427542

[76] Chang JP-C Lai M-C Chou M-C Shang C-Y Chiu Y-N Tsai W-C Maternal and family processes in different subgroups of youth with autism spectrum disorder. J Abnormal Child Psychol (2019) 47:177–94. 10.1007/s10802-018-0404-z 29417447

[77] Swanson JM Kraemer HC Hinshaw SP Arnold LE Conners CK Abikoff HB Clinical relevance of the primary findings of the MTA: success rates based on severity of ADHD and ODD symptoms at the end of treatment. J Am Acad Child Adolesc Psychiatry (2001) 40:168–79. 10.1097/00004583-200102000-00011 11211365

[78] Gau S-F Suen Soong W-T Psychiatric comorbidity of adolescents with sleep terrors or sleepwalking: a case-control study. Aust New Z J Psychiatry (1999) 33:734–9. 10.1080/j.1440-1614.1999.00610.x 10544999

[79] Fan LY Shang CY Tseng WYI Gau SSF Chou TL Visual processing as a potential endophenotype in youths with attention-deficit/hyperactivity disorder: A sibling study design using the counting Stroop functional MRI. Hum Brain Mapp (2018) 39:3827–35. 10.1002/hbm.24214 PMC6866550 29749060

[80] Lin H-Y Cocchi L Zalesky A Lv J Perry A Tseng W-YI Brain–behavior patterns define a dimensional biotype in medication-naïve adults with attention-deficit hyperactivity disorder. Psychol Med (2018) 48:2399–408. 10.1017/S0033291718000028 29409566

[81] Conners K Continuous performance test user’s manual. Toronto, Canada: Multi-Health systems (1995).

[82] Egeland J Kovalik-Gran I Validity of the factor structure of Conners’ CPT. J Attention Disord (2010) 13:347–57. 10.1177/1087054709332477 19448149

[83] Conners CK Conner’s Rating Scales-Revised User’s Manual. Toronto, Canada: Multi-Health Systems (1997).

[84] Gianarris WJ Golden CJ Greene L The Conners’parent Rating Scales: A Critical Review Of The Literature. Clin Psychol Rev (2001) 21:1061–93. 10.1016/S0272-7358(00)00085-4 11584516

[85] Conners CK Sitarenios G Parker JD Epstein JN Revision and restandardization of the Conners Teacher Rating Scale (CTRS-R): factor structure, reliability, and criterion validity. J Abnormal Child Psychol (1998) 26:279–91. 10.1023/A:1022606501530 9700520

[86] Conners CK Sitarenios G Parker JD Epstein JN The revised Conners’ Parent Rating Scale (CPRS-R): factor structure, reliability, and criterion validity. J Abnormal Child Psychol (1998) 26:257–68. 10.1023/A:1022602400621 9700518

[87] Rumelhart DE Hinton GE Williams RJ Learning internal representations by error propagation. No. ICS-8506. California Univ San Diego La Jolla Inst for Cognitive Science (1985).

[88] Schmidhuber J Deep learning in neural networks: An overview. Neural Networks (2015) 61:85–117. 10.1016/j.neunet.2014.09.003 25462637

[89] LeCun Y Bengio Y Hinton G Deep learning. Nature (2015) 521:436–44. 10.1038/nature14539 26017442

[90] Hertz JA Introduction to the theory of neural computation. CRC Press (2018).

[91] Glorot X Bordes A Bengio Y Deep Sparse Rectifier Neural Networks. Proceedings of the Fourteenth International Conference on Artificial Intelligence and Statistics, PMLR (2011) 15:315–23.

[92] Nasrabadi NM Pattern recognition and machine learning. J Electronic Imaging (2007) 16:049901. 10.1117/1.2819119

[93] Cortes C Vapnik V Support-vector networks. Mach Learn (1995) 20:273–97. 10.1007/BF00994018

[94] Ruder S An overview of gradient descent optimization algorithms. arXiv [Preprint] (2016). Available at: https://arxiv.org/abs/1609.04747 (Accessed Sep 15, 2016).

[95] Rennie SJ Goel V Thomas S Annealed dropout training of deep networks. 2014 IEEE Spoken Language Technology Workshop (SLT), South Lake Tahoe, NV (2014), p. 159–64. 10.1109/SLT.2014.7078567

[96] Caruana R Lawrence S Giles CL Overfitting in neural nets: Backpropagation, conjugate gradient, and early stopping. Advances in Neural Information Processing Systems 13 - Proceedings of the 2000 Conference, NIPS 2000. Neural information processing systems foundation (2001), p. 402–8.

[97] Yao Z Gholami A Keutzer K Mahoney M Large batch size training of neural networks with adversarial training and second-order information. arXiv [Preprint] (2018). Available at: https://arxiv.org/abs/1810.01021 (Accessed Oct 2, 2018).

[98] Keskar NS Mudigere D Nocedal J Smelyanskiy M Tang PTP On large-batch training for deep learning: Generalization gap and sharp minima. arXiv [Preprint] (2016). Available at: https://arxiv.org/abs/1609.04836 (Accessed Sep 15, 2016).

[99] Li M Zhang T Chen Y Smola AJ Efficient mini-batch training for stochastic optimization. Proceedings of the 20th ACM SIGKDD international conference on Knowledge discovery and data mining (KDD ’14). Association for Computing Machinery, New York, NY, USA (2014), p. 661–70. 10.1145/2623330.2623612

[100] Artetxe A Beristain A Grana M Predictive models for hospital readmission risk: A systematic review of methods. Comput Methods Programs Biomed (2018) 164:49–64. 10.1016/j.cmpb.2018.06.006 30195431

[101] Huang S Cai N Pacheco PP Narrandes S Wang Y Xu W Applications of support vector machine (SVM) learning in cancer genomics. Cancer Genom Proteom (2018) 15:41–51. 10.21873/cgp.20063 PMC5822181 29275361

[102] Karimi K Wuitchik DM Oldach MJ Vize PD Distinguishing species using GC contents in mixed DNA or RNA sequences. Evolution Bioinf (2018) 14. 10.1177/1176934318788866 PMC6052495 30038485

[103] Nahid A-A Kong Y Involvement of machine learning for breast cancer image classification: a survey. Comput Math Methods Med 2017 (2017) 2017:29. 10.1155/2017/3781951 PMC5804413 29463985

[104] Ioffe S Szegedy C Batch normalization: Accelerating deep network training by reducing internal covariate shift. arXiv [Preprint] (2015). Available at: https://arxiv.org/abs/1502.03167 (Accessed Feb 11, 2015).

[105] Halme AS Tannenbaum C Performance of a Bayesian Approach for Imputing Missing Data on the SF-12 Health-Related Quality-of-Life Measure. Value Health (2018) 21:1406–12. 10.1016/j.jval.2018.06.007 30502784

[106] Pedersen AB Mikkelsen EM Cronin-Fenton D Kristensen NR Pham TM Pedersen L Missing data and multiple imputation in clinical epidemiological research. Clin Epidemiol (2017) 9:157–66. 10.2147/CLEP.S129785 PMC5358992 28352203

[107] Keogh RH Seaman SR Bartlett JW Wood AM Multiple imputation of missing data in nested case-control and case-cohort studies. Biometrics (2018) 74:1438–49. 10.1111/biom.12910 PMC6481559 29870056

[108] Caye A Machado JD Rohde LA Evaluating parental disagreement in ADHD diagnosis: Can we rely on a single report from home? J Attention Disord (2017) 21:561–6. 10.1177/1087054713504134 24097846

[109] Orylska A Brzezicka A Racicka-Pawlukiewicz E Albinski R Sedek G Parent-Teacher Concordance in Rating Preschooler Difficulties in Behavioural and Cognitive Functioning and Their Dyadic Predicting of Fluid Intelligence. Polish psychol Bull (2016) 47:81–91. 10.1515/ppb-2016-0009

[110] Olson SL Davis-Kean P Chen M Lansford JE Bates JE Pettit GS Mapping the growth of heterogeneous forms of externalizing problem behavior between early childhood and adolescence: A comparison of parent and teacher ratings. J Abnormal Child Psychol (2018) 46:935–50. 10.1007/s10802-018-0407-9 PMC6124305 29488107

[111] Saad D Online algorithms and stochastic approximations. Online Learning in Neural Networks (1997 Workshop at the Newton Institute). The Newton Institute Series. Cambridge University Press, Cambridge (1998), p. 9–42.

