# Supplementary material for: A Deep Learning Approach for Missing Data Imputation of Rating Scales Assessing Attention-Deficit Hyperactivity Disorder
Source: Front Psychiatry. 2020 Jul 17;11:673. doi: 10.3389/fpsyt.2020.00673 (PMC7379397; doi:10.3389/fpsyt.2020.00673)

Supplementary Material

Supplementary Table 1 Imputation order and other details.

Order	Scales	Items	Title	Category	Numbers imputed
**1**	CTRS-R:S	10	Spiteful or vindictive	Oppositional	449
**2**	SNAP-IV-T	26	Is spiteful or vindictive	Oppositional	621
**3**	CTRS-R:S	6	Actively defies or refuses to comply with adults' requests	Oppositional	448
**4**	CTRS-R:S	20	Temper outbursts; explosive, unpredictable behavior	Oppositional	450
**5**	CTRS-R:S	2	Defiant	Oppositional	447
**6**	SNAP-IV-P	26	Is spiteful or vindictive	Oppositional	420
**7**	SNAP-IV-T	20	Argues with adults	Oppositional<br>Impulsivity	620
**8**	SNAP-IV-T	21	Actively defines or refuses adult requests or rules	Oppositional<br>Hyperactivity	620
**9**	SNAP-IV-T	12	Runs about or climbs excessively in situations where it is inappropriate	Hyperactivity	621

**10**	SNAP-IV-T	25	Is angry and resentful	Oppositional	620
**11**	CTRS-R:S	15	Argues with adults	Oppositional	448
**12**	SNAP-IV-T	19	Loses temper	Oppositional	621
**13**	SNAP-IV-T	11	Leaves seat in classroom or in other situations in which remaining seated is expected	Hyperactivity	620
**14**	CTRS-R:S	21	Runs about or climbs excessively in situations where it is inappropriate	Hyperactivity	450
**15**	CPRS-R:S	23	Leaves seat in classroom or in other situations in which remaining seated is expected	ADHD Index	213
**16**	SNAP-IV-T	24	Is touchy or easily annoyed by others	Oppositional	619
**17**	SNAP-IV-T	17	Loses things necessary for tasks or activities (eg, school assignments, pencils, books, tools, or toys)	Hyperactivity<br>Impulsivity	621
**18**	CTRS-R:S	11	Leaves seat in classroom or in other situations where remaining seated is expected	Hyperactivity	453
**19**	CTRS-R:S	17	Has difficulty waiting his/her turn	Hyperactivity	448
**20**	SNAP-IV-T	14	Is always "on the go" or acts as if driven by a motor	Hyperactivity	619
**21**	CTRS-R:S	24	Has difficulty playing or engaging in leisure activities quietly	Hyperactivity	449
**22**	CTRS-R:S	7	Is always "on the go" or acts as if driven by a motor	Hyperactivity	448

**23**	CTRS-R:S	28	Restless, always up and on the go	ADHD Index	450
**24**	SNAP-IV-P	11	Leaves seat in classroom or in other situations in which remaining seated is expected	Hyperactivity	420
**25**	CTRS-R:S	12	Fidgets with hands or feet or squirms in seat	ADHD Index	451
**26**	CPRS-R:S	9	Hard to control in malls or while grocery shopping	Hyperactivity	210
**27**	SNAP-IV-P	12	Runs about or climbs excessively in situations where it is inappropriate	Hyperactivity	417
**28**	SNAP-IV-T	13	Has difficulty playing or engaging in leisure activities quietly	Hyperactivity	622
**29**	SNAP-IV-T	22	Does things deliberately that annoy other people	Oppositional<br>Hyperactivity	620
**30**	CPRS-R:S	26	Has difficulty playing or engaging in leisure activities quietly	Hyperactivity	209
**31**	SNAP-IV-T	6	Avoids, expresses reluctance about, or has difficulties engaging in tasks that require sustained mental effort (such as schoolwork or homework)	Inattention	620
**32**	SNAP-IV-P	13	Has difficulty playing or engaging in leisure activities quietly	Hyperactivity	418
**33**	CTRS-R:S	13	Not reading up to par	Cognitive Problems/Inattention	450
**34**	CPRS-R:S	14	Runs about or climbs excessively in situations where it is inappropriate	Hyperactivity	209
**35**	SNAP-IV-P	17	Has difficulty waiting in lines or awaiting turn in games or group situations	Hyperactivity<br>Impulsivity	419

**36**	CPRS-R:S	22	Has difficulty waiting in lines or awaiting turn in games or group situations	Hyperactivity	210
**37**	SNAP-IV-T	10	Fidgets with hands or feet or squirms in seat	Inattention<br>Hyperactivity	619
**38**	CPRS-R:S	24	Deliberately does things that annoy other people	Oppositional	211
**39**	CPRS-R:S	16	Irritable	Oppositional	210
**40**	CTRS-R:S	26	Does not follow through on instructions and fails to finish schoolwork (not due to oppositional behavior or failure to understand instructions)	ADHD Index<br>Cognitive Problems/Inattention	452
**41**	SNAP-IV-T	23	Blames others for his or her mistakes or misbehavior	Oppositional	619
**42**	CTRS-R:S	5	Disturbs other children	ADHD Index	448
**43**	SNAP-IV-P	22	Does things deliberately that annoy other people	Oppositional<br>Hyperactivity	417
**44**	SNAP-IV-T	2	Has difficulty sustaining attention in tasks or play activities	Inattention	619
**45**	CTRS-R:S	18	Lacks interest in schoolwork	Cognitive Problems/Inattention	448
**46**	SNAP-IV-T	4	Does not follow through on instructions and fails to finish schoolwork or chores (not due to oppositional behavior or failure to understand instructions)	Inattention	621

**47**	SNAP-IV-T	18	Interrupts or intrudes on others (eg, butts into other’s conversation or games)	Impulsivity	623
**48**	SNAP-IV-P	14	Is always "on the go" or acts as if driven by a motor	Hyperactivity	417
**49**	CTRS-R:S	8	Poor in spelling	Cognitive Problems/Inattention	448
**50**	CPRS-R:S	4	Is always "on the go" or acts as if driven by a motor	Hyperactivity	211
**51**	CTRS-R:S	9	Cannot remain still	ADHD Index	447
**52**	SNAP-IV-P	18	Interrupts or intrudes on others (eg, butts into other's conversation or games)	Impulsivity	419
**53**	CTRS-R:S	23	Interrupts or intrudes on others (eg, butts into other's conversation or games)	ADHD Index	449
**54**	CTRS-R:S	27	Excitable, impulsive	ADHD Index<br>Hyperactivity	449
**55**	CTRS-R:S	3	Restless in the "squirmy" sense	Hyperactivity	450
**56**	SNAP-IV-T	16	Blurts out answers to questions before the questions have been completed	Impulsivity	620
**57**	CTRS-R:S	22	Poor in arithmetic	Cognitive Problems/Inattention	450
**58**	SNAP-IV-P	23	Blames others for his or her mistakes or misbehavior	Oppositional	417
**59**	CTRS-R:S	4	Forgets things he/she has already learned	Cognitive Problems/Inattention	447

**60**	SNAP-IV-P	25	Is angry and resentful	Oppositional	417
**61**	CPRS-R:S	25	Does not follow through on instructions and fails to finish schoolwork, chores or duties in the workplace (note due to oppositional behavior or failure to understand instructions)	ADHD Index<br><br>Cognitive Problems/Inattention	211
**62**	SNAP-IV-T	5	Has difficulty organizing tasks and activities	Inattention	625
**63**	CTRS-R:S	19	Distractibility or attention span a problem	ADHD Index	449
**64**	CTRS-R:S	14	Short attention span	ADHD Index	451
**65**	CTRS-R:S	25	Fails to finish things he/she starts	ADHD Index	450
**66**	CPRS-R:S	3	Difficulty doing or completing homework	Cognitive Problems/Inattention	211
**67**	CPRS-R:S	18	Restless in the "squirmy" sense	Hyperactivity	209
**68**	CPRS-R:S	2	Angry and resentful	Oppositional	211
**69**	CPRS-R:S	8	Fails to complete assignments	Cognitive Problems/Inattention	211
**70**	CTRS-R:S	1	Inattentive, easily distracted	ADHD Index	447
**71**	CPRS-R:S	13	Only attends if it is something he/she is very interested in	ADHD Index	210
**72**	CPRS-R:S	12	Needs close supervision to get through assignments	Cognitive Problems/Inattention	211

**73**	CPRS-R:S	7	Fidgets with hands or feet or squirms in seat	ADHD Index	210
**74**	CPRS-R:S	11	Loses temper	Oppositional	211
**75**	SNAP-IV-P	4	Does not follow through on instructions and fails to finish schoolwork, chores or duties in the workplace (note due to oppositional behavior or failure to understand instructions)	Inattention	415
**76**	CPRS-R:S	17	Avoids, expresses reluctance about, or has difficulties engaging in tasks that require sustained mental effort (such as schoolwork or homework)	ADHD Index<br><br>Cognitive Problems/Inattention	210
**77**	SNAP-IV-P	19	Loses temper	Oppositional	417
**78**	CPRS-R:S	20	Actively defies or refuses to comply with adults' requests	Oppositional	209
**79**	SNAP-IV-T	15	Talks excessively	Impulsivity	619
**80**	SNAP-IV-T	7	Loses things necessary for tasks or activities (eg, school assignments, pencils, books, tools, or toys)	Inattention	623
**81**	SNAP-IV-T	3	Does not seem to listen to what is being said to him or her	Inattention	619
**82**	SNAP-IV-P	6	Avoids, expresses reluctance about, or has difficulties engaging in tasks that require sustained mental effort (such as schoolwork or homework)	Inattention	415
**83**	CPRS-R:S	15	Distractibility or attention span a problem	ADHD Index	210
**84**	SNAP-IV-T	8	Is easily distracted by extraneous stimuli	Inattention	621
**85**	SNAP-IV-P	24	Is touchy or easily annoyed by others	Oppositional	417

**86**	SNAP-IV-T	9	Is forgetful in daily activities	Inattention	620
**87**	SNAP-IV-P	10	Fidgets with hands or feet or squirms in seat	Inattention<br>Hyperactivity	419
**88**	CPRS-R:S	21	Has trouble concentrating in class	ADHD Index<br>Cognitive Problems/Inattention	211
**89**	SNAP-IV-P	5	Has difficulty organizing tasks and activities	Inattention	415
**90**	SNAP-IV-P	3	Does not seem to listen to what is being said to him or her	Inattention	415
**91**	SNAP-IV-P	21	Actively defines or refuses adult requests or rules	Oppositional<br>Hyperactivity	418
**92**	CPRS-R:S	19	Gets distracted when given instructions to do something	ADHD Index	210
**93**	SNAP-IV-P	1	Fails to give close attention to details or makes careless mistakes in schoolwork or other activities	Inattention	416
**94**	CPRS-R:S	27	Easily frustrated in efforts	ADHD Index	210
**95**	CPRS-R:S	6	Argues with adults	Oppositional	210
**96**	SNAP-IV-P	2	Has difficulty sustaining attention in tasks or play activities	Inattention	415
**97**	CPRS-R:S	1	Inattentive, easily distracted	ADHD Index	210

**98**	SNAP-IV-P	8	Is easily distracted by extraneous stimuli	Inattention	415
**99**	SNAP-IV-T	1	Fails to give close attention to details or makes careless mistakes in schoolwork or other activities	Inattention	619
**100**	SNAP-IV-P	20	Argues with adults	Oppositional<br>Impulsivity	417
**101**	SNAP-IV-P	9	Is forgetful in daily activities	Inattention	417
**102**	SNAP-IV-P	16	Blurts out answers to questions before the questions have been completed	Impulsivity	419
**103**	CPRS-R:S	5	Short attention span	ADHD Index	210
**104**	CTRS-R:S	16	Only pats attention to things he/she is really interested in	ADHD Index	450
**105**	SNAP-IV-P	7	Loses things necessary for tasks or activities (eg, school assignments, pencils, books, tools, or toys)	Inattention	415
**106**	SNAP-IV-P	15	Talks excessively	Impulsivity	418
**107**	CPRS-R:S	10	Messy or disorganized at home or school	ADHD Index	210

Supplementary Table 2 Full data classification accuracy comparison of deep learning imputation (our method) with non-deep learning different imputation methods.

**Method**	ADHD/TD Classification Accuracy
The original group with the completed dataset, DL imputation	0.8931 ± 0.0215
ES=100, BS=Batch, D=0.25, DL imputation	0.8859 ± 0.0142
ES=100, BS=Batch, D=0.2, DL imputation	0.8987 ± 0.0139
ES=100, BS=Batch, D=0.5, DL imputation	0.8954 ± 0.0164
ES=100, BS=8, D=0.25, DL imputation	0.8918 ± 0.0113
ES=10, BS=Batch, D=0.25, DL imputation	0.8885 ± 0.0118
ES=10, BS=Batch, D=0.5, DL imputation	0.899 ± 0.0121
ES=10, BS=8, D=0.25, DL imputation	0.8921 ± 0.0109
ES=10, BS=Stochastic, D=0.25, DL imputation	0.9046 ± 0.0159
Interpolate imputation	0.77±0.05
Mean imputation	0.75±0.03
Multiple imputations	0.76±0.06

Results presented as $means \pm standard\ deviations$. All results were run 10-fold cross-validation; ES, earlystopping patience; BS, batch size; D, dropout rate; DL, deep learning.

Supplementary Table 3 Classification accuracy of attention-deficit/hyperactivity disorder (ADHD) vs. typically developing (TD) controls in models with different combinations of hyper-parameters (early stopping, batch size, and dropout rate). The same analysis like Table 2 (manuscript) but removing ODD symptom items.

**Method**	ADHD/TD Classification Accuracy
ES=100, BS=Batch, D=0.2, remove ODD items	0.8956±0.01
ES=100, BS=Batch, D=0.25, remove ODD items	0.8955±0.01
ES=100, BS=Batch, D=0.5, remove ODD items	0.9001±0.01
ES=100, BS=8, D=0.2, remove ODD items	0.8961±0.01
ES=100, BS=8, D=0.25, remove ODD items	0.8951±0.01
ES=100, BS=8, D=0.5, remove ODD items	0.8872±0.02
ES=10, BS= Batch, D=0.2, remove ODD items	0.9011±0.01
ES=10, BS= Batch, D=0.25, remove ODD items	0.8951±0.01
ES=10, BS= Batch, D=0.5, remove ODD items	0.8934±0.01
ES=10, BS= 8, D=0.2, remove ODD items	0.8959±0.02
ES=10, BS= 8, D=0.25, remove ODD items	0.8897±0.01
ES=10, BS= 8, D=0.5, remove ODD items	0.9003±0.01
ES=10, BS= Stochastic, D=0.25, remove ODD items	0.9022±0.01

Results presented as $means \pm standard\ deviations$. All results were run 10-fold cross-validation; ES, Early Stop; BS, Batch Size; D, Dropout rate.

Supplementary Table 4 details history for hyper-parameter combinations.

Please see Supplementary_table4.xlsx

Supplementary Table 5 Imputation order based on the rating scales that ODD symptoms excluded.

Order	Scales	Items	Category
**1**	CPRS-R:S	23	ADHD Index
**2**	CTRS-R:S	21	Hyperactivity
**3**	SNAP-IV-T	12	Hyperactivity
**4**	CTRS-R:S	13	Cognitive Problems/Inattention
**5**	CTRS-R:S	17	Hyperactivity
**6**	SNAP-IV-T	17	Hyperactivity<br>Impulsivity
**7**	CTRS-R:S	11	Hyperactivity
**8**	SNAP-IV-T	11	Hyperactivity
**9**	SNAP-IV-P	17	Hyperactivity<br>Impulsivity
**10**	SNAP-IV-T	22	Hyperactivity
**11**	CTRS-R:S	24	Hyperactivity
**12**	SNAP-IV-P	11	Hyperactivity
**13**	SNAP-IV-P	12	Hyperactivity
**14**	CTRS-R:S	12	ADHD Index
**15**	CTRS-R:S	7	Hyperactivity
**16**	SNAP-IV-T	14	Hyperactivity
**17**	CPRS-R:S	9	Hyperactivity
**18**	SNAP-IV-T	13	Hyperactivity
**19**	CPRS-R:S	22	Hyperactivity
**20**	CTRS-R:S	28	ADHD Index
**21**	CTRS-R:S	26	ADHD Index, Cognitive Problems/Inattention
**22**	CPRS-R:S	14	Hyperactivity
**23**	CTRS-R:S	5	ADHD Index
**24**	SNAP-IV-T	18	Impulsivity
**25**	CTRS-R:S	8	Cognitive Problems/Inattention
**26**	CPRS-R:S	26	Hyperactivity
**27**	CTRS-R:S	18	Cognitive Problems/Inattention
**28**	CTRS-R:S	23	ADHD Index
**29**	CTRS-R:S	27	ADHD Index<br>Hyperactivity

**30**	SNAP-IV-T	4	Inattention
**31**	CTRS-R:S	22	Cognitive Problems/Inattention
**32**	SNAP-IV-P	14	Hyperactivity
**33**	SNAP-IV-T	6	Inattention
**34**	CTRS-R:S	3	Hyperactivity
**35**	SNAP-IV-P	13	Hyperactivity
**36**	CPRS-R:S	4	Hyperactivity
**37**	SNAP-IV-T	16	Impulsivity
**38**	SNAP-IV-T	7	Inattention
**39**	CTRS-R:S	9	ADHD Index
**40**	CTRS-R:S	25	ADHD Index
**41**	CPRS-R:S	18	Hyperactivity
**42**	CPRS-R:S	27	ADHD Index
**43**	SNAP-IV-T	10	Inattention<br>Hyperactivity
**44**	SNAP-IV-T	5	Inattention
**45**	CTRS-R:S	14	ADHD Index
**46**	SNAP-IV-P	8	Inattention
**47**	CPRS-R:S	13	ADHD Index
**48**	CTRS-R:S	4	Cognitive Problems/Inattention
**49**	SNAP-IV-P	7	Inattention
**50**	CPRS-R:S	3	Cognitive Problems/Inattention
**51**	CTRS-R:S	19	ADHD Index
**52**	CPRS-R:S	7	ADHD Index
**53**	SNAP-IV-P	4	Inattention
**54**	SNAP-IV-T	2	Inattention
**55**	SNAP-IV-T	15	Impulsivity
**56**	CPRS-R:S	25	ADHD Index, Cognitive Problems/Inattention
**57**	SNAP-IV-T	1	Inattention
**58**	SNAP-IV-P	2	Inattention
**59**	SNAP-IV-P	18	Impulsivity
**60**	SNAP-IV-P	3	Inattention
**61**	CPRS-R:S	8	Cognitive Problems/Inattention
**62**	SNAP-IV-T	3	Inattention

**63**	SNAP-IV-P	9	Inattention
**64**	SNAP-IV-P	16	Impulsivity
**65**	SNAP-IV-T	8	Inattention
**66**	SNAP-IV-P	1	Inattention
**67**	CPRS-R:S	17	ADHD Index, Cognitive Problems/Inattention
**68**	SNAP-IV-T	9	Inattention
**69**	SNAP-IV-P	6	Inattention
**70**	CPRS-R:S	12	Cognitive Problems/Inattention
**71**	CPRS-R:S	19	ADHD Index
**72**	SNAP-IV-P	5	Inattention
**73**	CPRS-R:S	1	ADHD Index
**74**	CTRS-R:S	16	ADHD Index
**75**	CPRS-R:S	10	ADHD Index
**76**	CTRS-R:S	1	ADHD Index
**77**	SNAP-IV-P	15	Impulsivity
**78**	CPRS-R:S	15	ADHD Index
**79**	SNAP-IV-P	10	Inattention<br>Hyperactivity
**80**	CPRS-R:S	21	ADHD Index, Cognitive Problems/Inattention
**81**	CPRS-R:S	5	ADHD Index

Supplementary Figure 1 ODD symptoms free results in different combinations of hyper-parameters. (A) discriminatory ability – the accuracy of the imputed dataset in classifying ADHD vs. typically-developing (TD) controls for each iteration. (B) predictive power – the accuracy of deep neural network (DNN) to impute candidate question's missing values for each iteration.

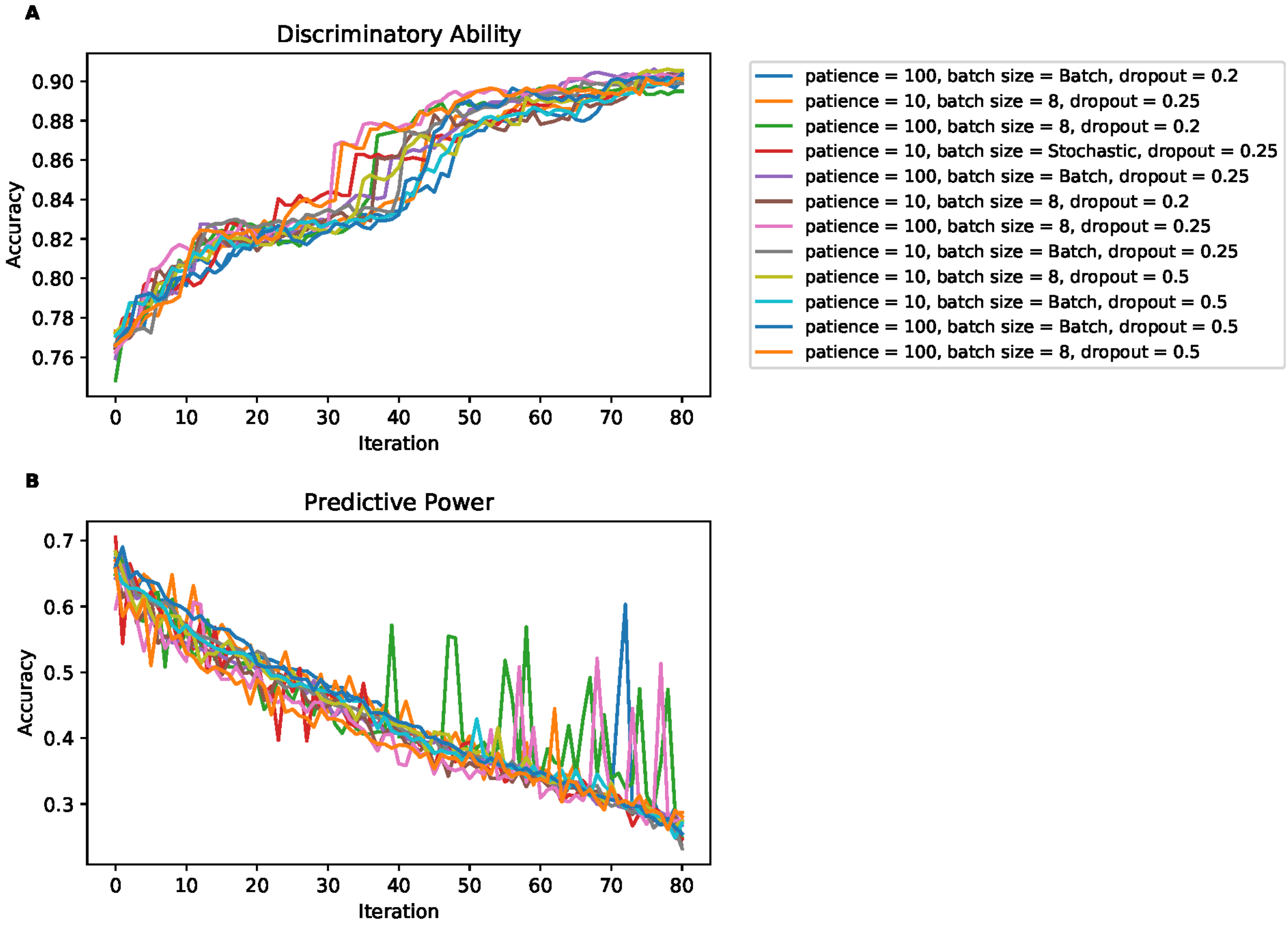

Supplement: Supplementary file 1 [file DataSheet_1.pdf]
